# Serum GPC3 and AKR1B10 as Diagnostic Biomarkers for Hepatitis E Virus–Associated Acute Liver Failure

**DOI:** 10.5152/tjg.2026.29017

**Published:** 2026-07-21

**Authors:** Guozhong Zhang, Xin Liu, Ying Wang, Kang Lu, Yang Wang

**Affiliations:** 1 Clinical Laboratory Center, Beijing YouAn Hospital, Capital Medical University, Beijing, China; 2 Clinical Laboratory Center, Beijing Likang Hospital, Beijing, China; 3 Department of Clinical Oncology, Beijing YouAn Hospital, Capital Medical University, Beijing, China

**Keywords:** Acute liver failure, AKR1B10, glypican-3, hepatitis E virus, hepatitis E virus–associated acute liver failure, risk factors

## Abstract

**Background/Aims:**

Hepatitis E virus (HEV)–associated acute liver failure (ALF) lacks reliable biomarkers for risk stratification at hospital admission. This study evaluated the diagnostic utility of serum glypican-3 (GPC3) and aldo-keto reductase family 1 member B10 (AKR1B10).

**Materials and Methods:**

Between May 2023 and May 2025, 231 adults with acute HEV infection were classified as HEV-ALF (n = 43) or HEV without ALF (n = 188). Serum GPC3 and AKR1B10 concentrations were measured using enzyme-linked immunosorbent assay. Associations were evaluated using Pearson correlation coefficient *r*; diagnostic performance was assessed by receiver operating characteristic curves, and independent predictors of ALF were identified using multivariable logistic regression.

**Results:**

Patients with HEV-ALF had lower albumin, triglyceride, and cholesterol levels and higher total bilirubin, prothrombin time, and international normalized ratio values than patients without ALF (all *P* < .05). GPC3 and AKR1B10 concentrations were markedly elevated in the HEV-ALF group (both *P* < .001) and were strongly correlated (*r* = 0.610, *P* < .001). The combination of GPC3 and AKR1B10 demonstrated superior diagnostic performance for identifying ALF, with an area under the curve (AUC) of 0.931, outperforming either GPC3 alone (AUC, 0.832) or AKR1B10 alone (AUC, 0.792). In multivariable logistic regression analysis, elevated GPC3 (odds ratio [OR], 2.138; 95% CI, 1.213-3.771) and AKR1B10 (OR, 2.304; 95% CI, 1.215-4.368) remained independent risk factors for ALF.

**Conclusion:**

Serum GPC3 and AKR1B10 were independent predictors of HEV-ALF and may facilitate risk stratification at hospital admission.

**Main Points**

- Serum glypican-3 (GPC3) and aldo-keto reductase family 1 member B10 (AKR1B10) were significantly higher in patients with hepatitis E virus–associated acute liver failure (HEV-ALF) than in those HEV infection without ALF.
- Both biomarkers were independent predictors of HEV-ALF with an odds ratios >2.
- The combination of GPC3 + AKR1B10 achieved receiver operating characteristic area under the curve of 0.931, outperforming either biomarkers alone.
- Elevated GPC3 and AKR1B10 levels reflect severe hepatocellular injury and aberrant regenerative signaling.
- Simple enzyme-linked immunosorbent assay quantification of these 2 serum proteins enables risk stratification at hospital admission in adults with HEV infection.

## Introduction

Acute liver failure (ALF) is characterized by acute hepatic necrosis, coagulopathy, encephalopathy, and multiorgan failure, most commonly caused by viral hepatitis or drug toxicity.^1^ Hepatitis E virus (HEV), the most common cause of acute viral hepatitis, is usually subclinical but remains a leading cause of ALF. Global HEV-related mortality is approximately 1%-2%; however, in developing regions 20%-40% of acute HEV infections progress to ALF, with risk reaching up to 50% among infected pregnant women.^2^^,^^3^ As an acute trigger of ALF, HEV infection has received increasing attention, highlighting the urgent need for biomarkers that enable early diagnosis and risk stratification to improve clinical management and outcomes in HEV-associated ALF (HEV-ALF).

Glypican-3 (GPC3), a membrane-anchored heparan-sulfate proteoglycan selectively overexpressed in hepatocellular carcinoma (HCC), serves as a diagnostic biomarker for HCC and is associated with fibrosis progression and risk of malignant transformation.^4^ Recently introduced as a therapeutic target for HCC immunotherapy, GPC3 has shown clinical promise; yet, its efficacy remains limited by tumor microenvironment–mediated suppression, necessitating further optimization.^5^ Aldo-keto reductase family 1 member B10 (AKR1B10) participates in inflammatory disease progression by modulating signal transduction, glucose–lipid metabolism, and oxidative stress. Elevated AKR1B10 has been reported in nonalcoholic fatty liver disease (NAFLD), hepatic fibrosis, and other liver injuries, indicating persistent hepatic damage and adverse outcomes.^6^ A meta-analysis further indicates that AKR1B10 demonstrates high diagnostic accuracy, with superior sensitivity and specificity for early-stage HCC.^7^

Consequently, identifying reliable predictors for accurate recognition of HEV-ALF is essential for precise risk stratification of patients with HEV infection, enhanced clinical surveillance, and optimized therapeutic strategies.

## Materials and Methods

### Study Population

Inclusion criteria: (i) confirmed hepatitis E according to European Association for the Study of the Liver (EASL) criteria: positive serum anti-HEV IgM or detectable HEV RNA, accompanied by elevated alanine aminotransferase (ALT)^8^^,^^9^; (ii) symptom onset within 4 weeks before enrollment; (iii) age ≥18 years; and (iv) written informed consent from the patient or legal guardian.

Exclusion criteria: (i) co-infection with hepatitis B or C virus; (ii) presence of alcoholic liver disease, NAFLD, drug-induced liver injury, autoimmune liver disease, or metabolic liver disorders; (iii) clinically, radiologically, or histologically confirmed cirrhosis or chronic hepatitis; (iv) chronic kidney disease stage 3 or higher, prior renal transplantation, or severe right-sided heart failure; (v) HCC or metastatic liver tumors; (vi) vascular liver disorders (e.g., Budd–Chiari syndrome and sinusoidal obstruction syndrome) or inherited metabolic liver diseases (e.g., Wilson’s disease, hemochromatosis, and α-1 antitrypsin deficiency); (vii) acute fatty liver of pregnancy; (viii) use of systemic immunosuppressants, chemotherapy, or biologic agents within the preceding 3 months; and (ix) missing key baseline data.

**Figure 1.**
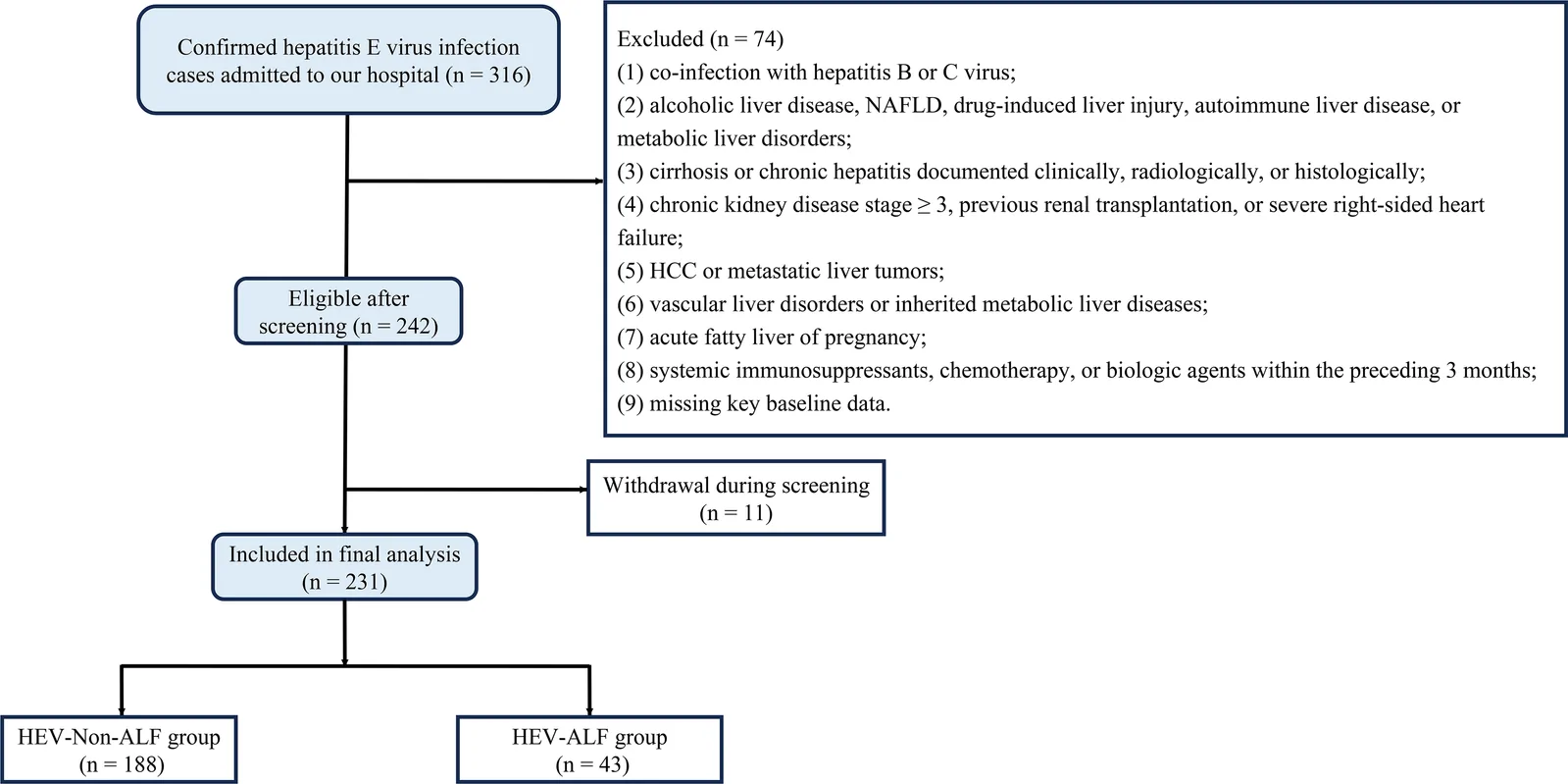
Flowchart of study participant screening in patients infected with hepatitis E virus.

This was a cross-sectional study with prospective, consecutive enrollment of 316 patients with HEV infection treated between April 2023 and May 2025. After rigorous screening, 231 patients were included (74 were excluded and 11 withdrew during the screening process). All biomarkers and standard laboratory parameters were measured simultaneously within 24 hours of admission. The study population comprised 137 men and 94 women, aged 47-73 years (mean age: 57.66 ± 6.40 years). The study protocol was approved by the ethics committee of Beijing YouAn Hospital, Capital Medical University (Approval No. 2023046; Date: April 24, 2023), and all participants provided written informed consent. No AI tools were used. The participant flow is shown in Figure 1.

### Hepatitis E Virus–Associated Acute Liver Failure Classification

Patients were stratified according to the presence or absence of HEV-ALF.^10^^,11^ HEV-ALF (n = 43) was defined as acute HEV infection within 4 weeks of onset accompanied by coagulopathy (international normalized ratio (INR) ≥ 1.5 or prothrombin time activity ≤ 40%) and hepatic encephalopathy (HE) of West Haven grade 2 or higher. Individuals not meeting these criteria were classified as HEV without ALF (HEV-non-ALF) (n = 188).

### Variables

Baseline data included demographic characteristics (gender, age, and body mass index), personal habits (smoking and alcohol use), comorbid conditions (hypertension, diabetes, and chronic kidney disease), and presenting features (fever, jaundice, abdominal pain, and nausea/vomiting). Laboratory parameters included hematologic indices (white blood cell count, hemoglobin, and platelet count), renal function markers (blood urea nitrogen and serum creatinine), liver function tests (albumin (ALB), aspartate aminotransferase, ALT, gamma-glutamyl transferase, and total bilirubin (TBIL)), lipid profile (triglycerides (TGs), total cholesterol (TC)), coagulation indices (prothrombin time (PT) and INR), and the inflammatory marker C-reactive protein.

### Serum GPC3 and AKR1B10 Assays

Whole blood was allowed to clot for 30 minutes at room temperature and then centrifuged at 1000 × g for 15 minutes; serum was stored for analysis. GPC3 (cat. no. ml104961) and AKR1B10 (cat. no. ml060485) were measured in duplicate using enzyme-linked immunosorbent assay (Shanghai Enzyme-linked Biotechnology, Shanghai, China) according to the manufacturer’s instructions. Absorbance was measured at 450 nm using a microplate reader (iMark, Bio-Rad, Hercules, California, USA). The assay demonstrated a coefficient of variation <10%, and mean values were used for analysis. All assays were performed by a technician blinded to clinical data.

### Statistical Analysis

Statistical analyses were performed using Statistical Package for the Social Sciences (SPSS) version 26.0 (IBM SPSS Corp.; Armonk, NY, USA). Continuous variables are presented as mean ± SD and were compared using the independent samples *t*-test when normally distributed; non-normally distributed variables are presented as median (interquartile range) and were compared using the Mann–Whitney *U* test. Categorical variables were compared using the chi-square (*χ*^2^) test. Pearson correlation analysis was used to assess the relationship between GPC3 and AKR1B10, as well as the correlations between these biomarkers and INR. Receiver operating characteristic (ROC) curve analysis and multivariable logistic regression were performed to evaluate the predictive performance of these biomarkers and to identify the risk factors for HEV-ALF. All statistical analyses were 2-sided, and a *P* value < .05 was considered significant.

## Results

### Baseline Characteristics and Univariate Analysis of Patients Infected with Hepatitis E Virus

Among the 231 patients with HEV infection, 43 (18.6%) developed HEV-ALF. Gender, age, and other baseline variables were comparable between groups (*P* > .05). Patients with HEV-ALF exhibited lower ALB, TG, and TC, accompanied by higher TBIL, PT, and INR, compared with patients without ALF (all *P* < .05) (Table 1).

### Comparison of Serum GPC3 and AKR1B10 Levels Between Patients Infected with Hepatitis E Virus with and Without Acute Liver Failure

Serum levels of GPC3 and AKR1B10 were both significantly elevated in the HEV-ALF group compared with those in the HEV-non-ALF group (both *P* < .001) (Table 2).

### Correlations of Serum Biomarkers with INR and Interbiomarker Correlation in Patients with Hepatitis E Virus–Associated Acute Liver Failure

**Figure 2.**
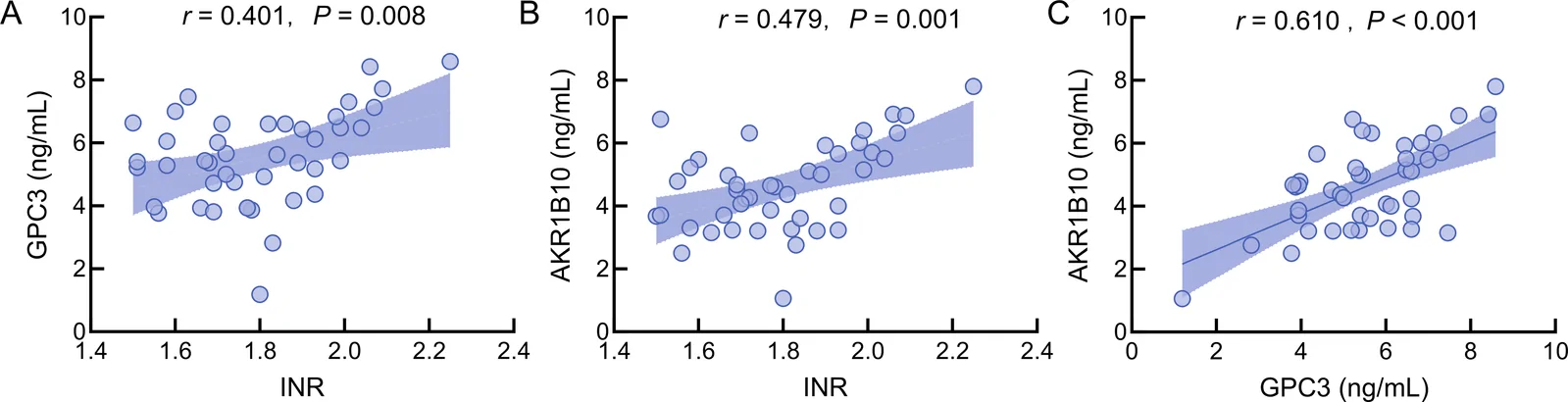
Correlations of serum GPC3 and AKR1B10 with INR and the interrelationship between thsese biomarkers in patients with HEV-ALF. (A) Pearson correlation between serum GPC3 levels and INR. (B) Pearson correlation between serum AKR1B10 levels and INR. (C) Pearson correlation between serum GPC3 and AKR1B10 levels. Shaded areas represent the 95% CI. AKR1B10, aldo-keto reductase family 1 member B10; GPC3, glypican-3; HEV-ALF, hepatitis E virus–associated acute liver failure.

Pearson correlation analysis demonstrated that serum GPC3 levels were significantly positively correlated with INR in the HEV-ALF group (*r* = 0.401, *P* = .008, Figure 2A). Similarly, serum AKR1B10 levels were significantly positively correlated with INR (*r* = 0.479, *P* = .001, Figure 2B). Furthermore, a significant positive correlation was observed between GPC3 and AKR1B10 levels (*r* = 0.610, *P* < .001, Figure 2C).

### Predictive Value of Serum GPC3 and AKR1B10 Levels for Hepatitis E Virus–Associated Acute Liver Failure Development in Patients with HEV Infection

**Figure 3.**
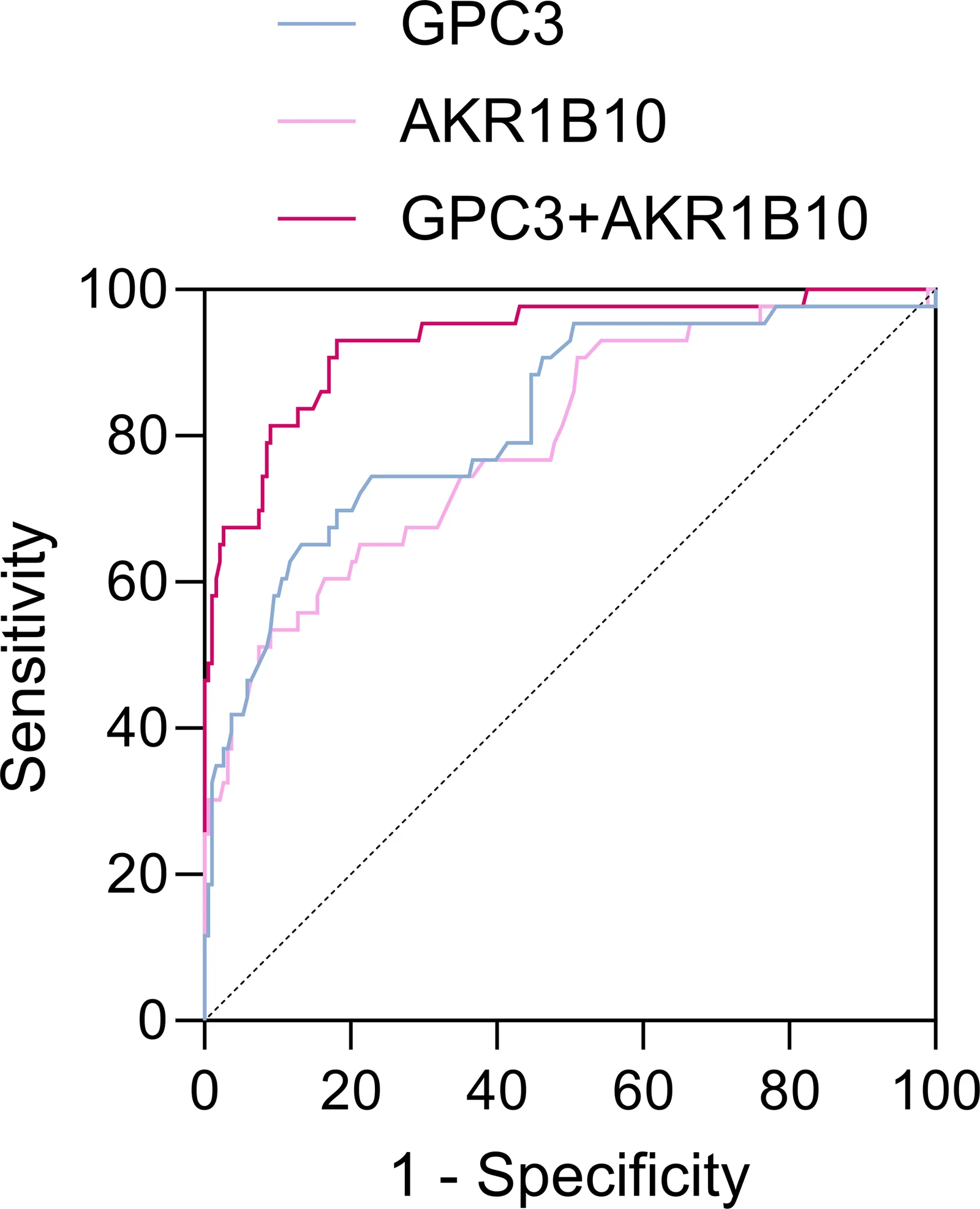
Receiver operating characteristic curves evaluating the predictive performance of GPC3 and AKR1B10 levels for the development of HEV-ALF in patients with HEV infection. AKR1B10, aldo-keto reductase family 1 member B10; GPC3, glypican-3; HEV-ALF, hepatitis E virus–associated acute liver failure.

ROC curve analyses demonstrated that GPC3 and AKR1B10 levels, as well as their combined model, predicted the development of HEV-ALF. The areas under the ROC curve were 0.832 (95% CI: 0.749-0.896) for GPC3, 0.792 (95% CI: 0.713-0.871) for AKR1B10, and 0.931 (95% CI: 0.885-0.977) for the combined model (Table 3, Figure 3).

### Multivariable Logistic Regression Analysis of Factors Influencing Progression to Hepatitis E Virus–Associated Acute Liver Failure

Using ALF occurrence as the dependent variable (absent = 0, present = 1), ALB, TG, TC, TBIL, PT, GPC3, and AKR1B10 (variables that demonstrated significance on univariate significance) were entered as covariates; INR was excluded because it is integral to the ALF definition (INR ≥ 1.5). Multivariable analysis identified elevated TBIL (odds ratio [OR] = 1.020, 95% CI: 1.006-1.034), prolonged PT (OR = 1.413, 95% CI: 1.151-1.734), elevated GPC3 (OR = 2.138, 95% CI: 1.213-3.771), and elevated AKR1B10 (OR = 2.304, 95% CI: 1.215-4.368) as independent risk factors for HEV-ALF (all *P* < .05). Conversely, higher ALB (OR = 0.829, 95% CI: 0.713-0.964), TG (OR = 0.122, 95% CI: 0.025-0.589), and TC (OR = 0.075, 95% CI: 0.015-0.383) were protective (Table 4).

## Discussion

HEV remains a major cause of both acute liver failure and acute-on-chronic liver failure. These conditions are characterized by intrahepatic immune cell infiltration and systemic elevations in proinflammatory cytokines and chemokines, highlighting immune dysregulation as a key mechanism underlying the development of HEV-ALF.^12^ In the present cohort, 18.6% of patients developed HEV-ALF, a proportion broadly consistent with the incidence of adverse outcomes reported in previous studies.^13^ These findings confirm that progression to ALF is a clinically important consequence of HEV infection. Reliable biomarkers are needed to identify patients at high risk at the time of admission and to guide prompt intervention.

GPC3, a membrane-anchored heparan sulfate proteoglycan, is selectively overexpressed in malignant hepatocytes in HCC. Through activation of oncogenic Wnt/β-catenin and phosphatidylinositol 3-kinase/protein kinase B (PI3K/AKT) signaling, GPC3 potently promotes tumor cell proliferation, migration, invasion, anti-apoptotic survival, and angiogenesis. It exerts a particularly strong driver effect on malignant transformation and disease progression in HBV-related HCC, positioning it as a key deleterious factor in hepatocarcinogenesis.^14^^-^^16^ AKR1B10, an nicotinamide adenine dinucleotide phosphate (NADPH)-dependent reductase induced by hepatic lipid-peroxidative stress, is markedly upregulated in NAFLD. Beyond serving as a critical circulating biomarker of disease progression across the NAFLD spectrum, AKR1B10 has been implicated in disease pathogenesis through its association with hepatic steatosis, insulin resistance, and dysregulated glucose and lipid metabolism. It therefore represents an important pathogenic contributor and serological indicator of liver injury and disease trajectory.^17^^-^^19^

Serum GPC3 and AKR1B10 levels were significantly higher in patients with HEV-ALF than in those without ALF, and both biomarkers were positively correlated with INR (*r* = 0.366 and 0.435, respectively). These findings suggest that elevated GPC3 and AKR1B10 levels are closely associated with the severity of coagulopathy and overall disease severity. This finding is most likely attributable to severe virus-induced hepatocellular injury and dysregulated regenerative responses. Increased GPC3 originates from aberrant regeneration and a latent malignant shift; functioning as a co-receptor in the Wnt/β-catenin, it fosters β-catenin activation and excessive hepatocyte proliferation—mechanisms already implicated in fibrogenesis and carcinogenesis.^20^ The elevation of AKR1B10 reflects intense inflammation, metabolic derangement, and compensatory proliferation. By modulating lipid metabolism and oxidative stress pathways, it aggravates hepatocyte dysfunction, mirroring its documented ability to potentiate injury and apoptosis resistance in nonalcoholic steatohepatitis.^21^

Multivariable logistic regression identified lower ALB, TG, and TC levels, along with higher TBIL and PT, as independent predictors of ALF in HEV infection, consistent with previous reports.^22^^,^^23^ Within the HEV-ALF group, GPC3 and AKR1B10 levels were positively correlated. This finding may reflect synergistic activation of a pathogenic feed-forward loop: Wnt/β-catenin signaling driven by GPC3 may upregulate AKR1B10 expression, whereas AKR1B10-mediated lipid metabolic reprogramming may further amplify Wnt pathway activity, Collectively, these processes may exacerbate hepatocellular metabolic dysregulation, aberrant proliferation, and apoptotic imbalance, thereby fueling disease progression. Experimental data support Wnt/β-catenin–dependent metabolic reprogramming as a driver of hepatocyte dysfunction; Wang et al^24^ demonstrated that activation of Wnt/β-catenin promotes de novo lipogenesis and suppresses fatty acid oxidation via Akt/mTOR signaling, while physically interacting with mTOR to aggravate high-fat-diet-induced hepatic steatosis, thereby providing mechanistic evidence that dysregulation of this pathway impairs hepatic metabolism. For the first time, the present study proposes combined serum GPC3 and AKR1B10 measurement as a tool for predicting ALF risk in patients with HEV infection, offering a novel strategy for early risk stratification and clinical intervention.

This study has several limitations. First, the HEV-ALF group had a relatively small sample size, and the single-center, single-etiology design limits the generalizability of the present findings. Stratified analyses were underpowered, highlighting the need for multicenter studies with larger cohorts to enhance reliability and broaden applicability. Second, the specific molecular mechanisms of GPC3 and AKR1B10 in HEV-ALF remain unclear Future studies should use cellular and animal models to further investigate their mechanistic pathways and interactive relationships. Third, although GPC3, AKR1B10, and standard laboratory parameters were measured simultaneously within 24 hours of admission, all assessments were performed at a single time point at enrollment. Therefore, this study lacked dynamic monitoring and was unable to determine whether changes in these biomarkers precede, coincide with, or follow alterations in conventional indices. In addition, dynamic trajectories could not be evaluated, nor could the optimal predictive time window be defined. Prospective longitudinal studies are warranted to clarify the dynamic changes in these biomarkers over the course of the disease and to determine whether they provide additional clinical value beyond standard laboratory testing. Fourth, the definition of HEV-ALF used in this study required HE of West Haven grade 2 or higher, which may have excluded patients with mild grade 1 encephalopathy. Whether GPC3 and AKR1B10 demonstrate similar predictive performance in patients with earlier or subtler HE manifestations warrants future investigation. Fifth, whether GPC3 and AKR1B10 are specific biomarkers for HEV infection remains to be determined. Their elevation may reflect shared pathophysiological processes common to various liver diseases, including hepatocellular injury, dysregulated regeneration, and oxidative stress. Their discriminatory value and whether the established cutoff values are specific to HEV-ALF across distinct etiologies such as drug-induced liver injury or HBV-related ALF require prospective external validation in independent multicenter datasets.

In summary, elevated serum GPC3 and AKR1B10 are independent risk factors for HEV-ALF, and their combined assessment facilitates early identification of high-risk patients at admission, providing a novel evidence-based tool for targeted clinical intervention.

## Figures and Tables

**Table 1.** Baseline Characteristics and Univariate Analysis of Patients Infected with Hepatitis E Virus

Indicator	HEV-non-ALF Group (n = 188)	HEV-ALF Group (n = 43)	*t*/*Z*/*χ*^2^	*P*
Gender [n (%)]				
Male	109 (57.98)	28 (65.12)	0.739*	.390
Female	79 (42.02)	15 (34.88)		
Age [*M* (*P*_25_, *P*_75_), years]	57 (52, 62)	59 (53, 64)	−1.114ˆ	.265
BMI (kg/m^2^, 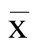 ± *s*)	22.24 ± 2.07	22.84 ± 2.13	−1.698^#^	.091
Smoking history [n (%)]	62 (32.98)	16 (37.21)	0.280*	.597
Alcohol consumption history [n (%)]	98 (52.13)	21 (48.84)	0.152*	.697
Comorbidities [n (%)]				
Hypertension	139 (73.94)	34 (79.07)	0.490*	.484
Diabetes mellitus	68 (36.17)	18 (41.86)	0.485*	.486
Chronic kidney disease	17 (9.04)	6 (13.95)	0.941*	.332
Clinical manifestations [n (%)]				
Fever	53 (28.19)	18 (41.86)	3.071*	.080
Jaundice	154 (81.91)	33 (76.74)	0.607*	.436
Abdominal pain	60 (31.91)	17 (39.53)	0.914*	.339
Nausea/vomiting	136 (72.34)	29 (67.44)	0.411*	.521
WBC [*M* (*P*_25_, *P*_75_), ×10^9^/L]	7.0 (6.1, 7.8)	7.0 (6.4, 7.9)	−0.597ˆ	.550
Hb (g/dL, 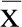 ± *s*)	13.76 ± 1.38	13.44 ± 1.52	1.337#	.183
PLT (×10^9^/L, 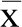 ± *s*)	153.43 ± 27.22	149.86 ± 30.43	0.759#	.449
BUN (mmol/L, 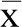 ± *s*)	5.82 ± 1.17	6.10 ± 1.25	−1.364#	.174
Scr (μmol/L, 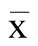 ± *s*)	69.86 ± 10.42	71.70 ± 11.23	−1.030#	.304
ALB [*M* (*P*_25_, *P*_75_), g/L]	32 (28, 36)	28 (25, 30)	−5.344ˆ	<.001
AST (U/L, 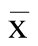 ± *s*)	218.20 ± 63.18	223.47 ± 71.98	−0.480#	.632
ALT (U/L, 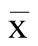 ± *s*)	297.76 ± 86.85	312.74 ± 92.02	−1.009#	.314
TG (mmol/L, 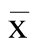 ± *s*)	2.26 ± 0.49	1.78 ± 0.32	6.125#	<.001
TC (mmol/L, 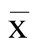 ± *s*)	2.88 ± 0.54	2.39 ± 0.40	5.608#	<.001
CRP [*M* (*P*_25_, *P*_75_), mg/dL]	12.1 (8.6, 14.4)	12.3 (8.8, 15.0)	−0.750ˆ	.453
GGT (U/L, 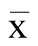 ± *s*)	149.85 ± 39.41	152.44 ± 42.15	−0.385#	.701
TBIL (μmol/L, 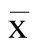 ± *s*)	181.85 ± 49.35	229.40 ± 50.45	−5.676#	<.001
PT [*M* (*P*_25_, *P*_75_), s]	18 (16, 20)	20 (19, 25)	−5.219ˆ	<.001
INR [*M* (*P*_25_, *P*_75_)]	1.08 (0.95, 1.18)	1.78 (1.67, 1.93)	−10.226ˆ	<.001

ALB, albumin; ALT, alanine aminotransferase; AST, aspartate aminotransferase; BMI, body mass index; BUN, blood urea nitrogen; CRP, C-reactive protein; GGT, gamma-glutamyl transferase; Hb, hemoglobin; INR, International Normalized Ratio; PLT, platelet count; PT, prothrombin time; Scr, serum creatinine; TBIL, total bilirubin; TC, total cholesterol; TG, triglyceride; WBC, white blood cell count.

*χ2 value.

^ˆ^*Z*-value.

^#^*t*-value.

**Table 2.** Comparison of Serum GPC3 and AKR1B10 Levels Between the 2 Groups of Patients with Hepatitis E Virus

Indicator	n	GPC3 (ng/mL)	AKR1B10 (ng/mL)
HEV-non-ALF group	188	3.81 ± 1.18	3.24 ± 0.98
HEV-ALF group	43	5.53 ± 1.50	4.62 ± 1.40
*t*		−8.161	−7.702
*P*		<.001	<.001

AKR1B10, aldo-keto reductase family 1 member B10; GPC3, glypican-3; HEV-ALF, hepatitis E virus–associated acute liver failure.

**Table 3.** Predictive Performance of Glypican-3 and Aldo-Keto Reductase Family 1 Member B10 for Acute Liver Failure Development in Patients with Hepatitis E Virus Infection

Indicator	Cutoff	AUC	Sensitivity	Specificity	*P*	95% Cl
GPC3	4.90	0.832	65.12	86.70	<.001	0.749-0.896
AKR1B10	5.92	0.792	53.49	90.96	<.001	0.713-0.871
GPC3 + AKR1B10	–	0.931	93.02	81.91	<.001	0.885-0.977

AKR1B10, aldo-keto reductase family 1 member B10; AUC, area under the curve; GPC3, glypican-3; HEV-ALF, hepatitis E virus–associated acute liver failure.

**Table 4.** Multivariable Logistic Regression Analysis of Factors Associated with Acute Liver Failure Development in Patients with Hepatitis E Virus Infection

Indicator	*β*	SE	Walds	*P*	OR	95% Cl
ALB	−0.188	0.077	5.968	.015	0.829	0.713~0.964
TG	−2.103	0.803	6.856	.009	0.122	0.025~0.589
TC	−2.595	0.834	9.686	.002	0.075	0.015~0.383
TBIL	0.020	0.007	7.878	.005	1.020	1.006~1.034
PT	0.346	0.105	10.926	.001	1.413	1.151~1.734
GPC3	0.760	0.289	6.896	.009	2.138	1.213~3.771
AKR1B10	0.835	0.326	6.541	.011	2.304	1.215~4.368

AKR1B10, aldo-keto reductase family 1 member B10; ALB, albumin; GPC3, glypican-3; OR, odds ratio; PT, prothrombin time; TBIL, total bilirubin; TC, total cholesterol; TG, triglyceride.

## Data Availability

The data that support the findings of this study are available on request from the corresponding author.
